# eHealth literacy assessment as a promoter of user adherence in using digital health systems and services. A case study for balance physiotherapy in the TeleRehaB DSS project

**DOI:** 10.3389/fdgth.2025.1535582

**Published:** 2025-07-31

**Authors:** Konstantinos Georgas, Konstantinos Bromis, Theodoros P. Vagenas, Olympia Giannakopoulou, Nikolaos Vasileiou, Ioannis Kouris, Maria Haritou, George K. Matsopoulos

**Affiliations:** Biomedical Engineering Laboratory, Institute of Communication and Computer Systems, NTUA, Athens, Greece

**Keywords:** digital health, eHealth literacy, health technology, mobile health (mHealth), patient adherence, telerehabilitation

## Abstract

Improving patient adherence and compliance with digital health interventions requires the creation of eHealth literacy resources. This study examines the creation and application of a novel eHealth literacy tool for home-based balance physiotherapy as part of the TeleRehaB DSS project. This tool evaluates patients’ digital literacy, in particular their ability to use the Internet of Things (IoT), Augmented Reality (AR) and smart device technologies. The tool addresses the challenge of low treatment adherence by utilizing models to monitor compliance in real time and adjust treatment recommendations accordingly. The TeleRehaB DSS integrates this literacy tool to maximize resource allocation and improve patient engagement. Testing and validation has shown the system’s ability to improve therapeutic outcomes and increase patient involvement. This strategy not only addresses the real-world difficulties of implementing digital health systems, but also advances the growing body of knowledge on improving treatment adherence through customized digital literacy assessments. When developing effective health technologies, the capabilities of users must be taken into account, especially for older people or those with limited digital literacy, as this study highlights.

## 1 Introduction

Digital technologies have become a critical component of patient-centered care in the dynamic healthcare landscape, enabling innovative approaches to diagnosis, treatment and rehabilitation. A key factor in this transformation is health literacy, which is the ability of an individual to understand and apply health information effectively to make decisions about their care (1). The ability to find, evaluate and use health information from electronic sources is referred to as eHealth literacy, which is a subset of health literacy (2). EHealth literacy is essential for patient participation and adherence as the use of digital health interventions increases, especially for populations that manage chronic diseases or undergo rehabilitation (3).

Despite the obvious advantages of digital health technologies, differences in the digital literacy of the population remain a major obstacle to their widespread adoption. Navigating digital health systems can be difficult for many patients, especially the elderly (4). This can lead to non-adherence to treatment programs and suboptimal health outcomes. Research has repeatedly shown that people with low eHealth literacy are less likely to participate in digital health interventions, which has a direct impact on their ability to manage their health successfully (5). Long-term patient engagement is critical to a successful recovery in rehabilitation, so this issue is of particular concern in this regard (6). To ensure more equitable access to these technologies, there is therefore an urgent need for apps that not only provide digital health services, but also assess and improve the digital literacy of users.

Assessing users’ eHealth literacy and digital skills is the main goal of the eHealth Literacy App created as part of the TeleRehaB DSS project. This assessment serves as the basis for customizing interventions in home-based balance physiotherapy programs. In particular, the app evaluates patients’ competency with Internet of Things (IoT), augmented reality (AR) and smart device technologies, enabling medical practitioners to customize treatment regimens according to patients’ degree of digital literacy. In addition, it offers actionable insights that let doctors modify treatments as necessary during real-time patient monitoring, guaranteeing that interventions are available and efficient for all users with varying technological capabilities. According to research, lack of digital literacy frequently leads to lower patient engagement and worse health outcomes, so this is crucial (7, 8).

The app’s capacity to incorporate eHealth literacy evaluations into the therapeutic process guarantees individualized treatment plans from the beginning improving adherence and health outcomes. This is where the significance of the study lies. The app addresses health equity issues by tackling digital literacy early, increasing the inclusion and accessibility of digital health technologies for users of all levels of technological skills. This is consistent with research in the larger body of literature showing that a major obstacle to accessing healthcare is a lack of digital literacy that exacerbates disparities (9). This strategy is an important step in making sure that technology supports care rather than hinders it, especially as digital health solutions proliferate in the medical field.

## 2 Related works

With the increasing reliance on mobile and web platforms for healthcare, eHealth literacy has emerged as a critical element in shaping digital health interventions. Tools such as the eHealth Literacy Scale (eHEALS) are commonly used to assess an individual’s ability to seek, understand and use health information from digital sources. However, the fact that eHEALS mostly emphasizes perceived ability over actual performance, limits its ability to accurately represent users’ actual digital abilities. This is a major criticism of the system. To obtain a more complete picture of eHealth literacy, recent research suggests that it is necessary to combine self-reported measures with tools that assess real-world performance (2, 10, 11).

Pavan et al. (12) investigated the effectiveness of two tele-rehabilitation (TR) models aimed at improving recovery from subacute upper limb disability following stroke. While the other cohort used non-robotic techniques, the first cohort received robot-assisted TR. Both groups showed remarkable progress in their cognitive and motor abilities, although the motor outcomes of the non-robotic group were slightly better. This suggests that despite the potential of robotic systems, conventional non-robotic methods can still achieve better results in certain rehabilitation settings.

Bok et al. (13) studied the effectiveness of home-based high-tech rehabilitation programs for stroke patients that focused on interventions such as virtual reality (VR), robotic devices and game-based techniques. According to the results, VR-based rehabilitation outperformed robotic and game-based interventions in terms of improving physical function, especially in upper limb recovery. This demonstrates the potential of immersive virtual reality technologies in rehabilitation programs conducted at home.

Jaramillo-Isaza et al. (14) implemented a comprehensive analysis of wearable sensors and artificial intelligence in tele-rehabilitation and presented how these technologies can enhance remote rehabilitation initiatives. Clinicians were able to make dynamic adjustments to treatment protocols using wearable devices that continuously monitored patients’ movements and provided real-time feedback. In home-based rehabilitation, this strategy significantly improved patient’s adherence and results, particularly for patients with mobility impairments.

Xie and Mo (15) conducted a meta-analysis comparing the Digital Health Literacy Instrument (DHLI) and the eHealth Literacy Scale (eHEALS) in the context of tele-rehabilitation of older adults. Their results showed that although both instruments measure perceived eHealth literacy well, the DHLI is better at identifying the practical digital skills required to successfully use tele-rehabilitation platforms. This research emphasizes the importance of using performance-based assessments to ensure that patients can interact with complicated digital health environments, which ultimately increases the effectiveness of tele-rehabilitation treatments.

## 3 Materials and methods

**Figure 1:**
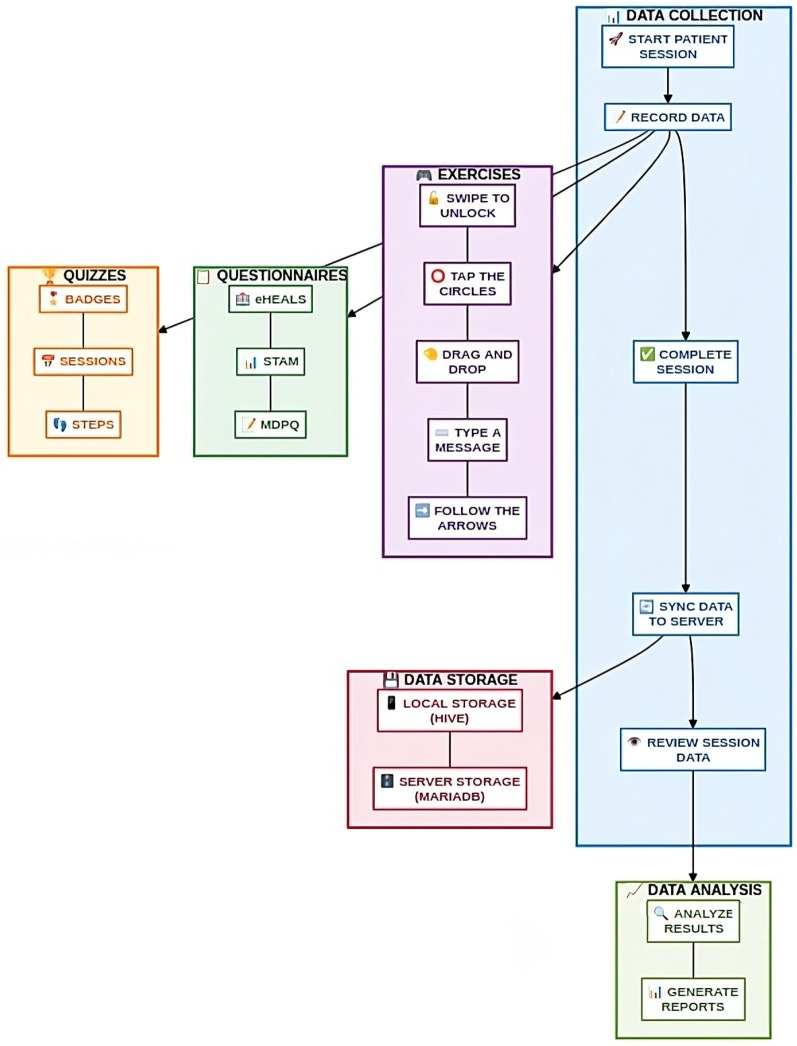
The Workflow diagram of the eHealth Literacy App.

The eHealth Literacy App was developed using a comprehensive methodological approach that addresses both technical and user-centered requirements. Before detailing the individual components, it is important to understand the overall structure and workflow of the app. The app operates in dual-mode, incorporating the patient mode for clinical use with specific patient data and session tracking, and the demo mode for training or demonstration purposes without patient data requirements. Figure 1 illustrates the overall workflow of the app and shows how the user progresses through the stages of data collection, exercise-questionnaires-quizzes completion and data synchronization. This workflow forms the foundation for all technical implementation decisions described in the following subsections.

### 3.1 System architecture

To guarantee cross-platform functionality, data security and effective synchronization, a strong technology stack was used during the development of the eHealth Literacy App. The Flutter framework, which enables the development of natively compiled applications for iOS and Android platforms from a single codebase, was used to implement the frontend (16). This method streamlines development and improves usability. .NET Core was chosen for the backend implementation as it has excellent performance characteristics, built-in security features, and efficient resource utilization with high concurrent load (17). The architectural flexibility of the framework allows the implementation of robust authentication protocols and encrypted data transfers, which are essential for handling sensitive patient data, while the modular design facilitated integration with healthcare-specific data processing modules. REST APIs ensure reliable and effective data exchange by enabling seamless communication between the frontend and backend, supporting the real-time synchronization required for patient monitoring and assessment.

### 3.2 Data management and security protocols

To maintain availability and persistence of data, the application uses a dual-database architecture. The main server-side database MariaDB is used to safely store exercise results, session data and patient records. Hive, a lightweight NoSQL database, is utilized for local storage within the application to guarantee offline functioning, automatically synchronizing all data upon reconnection (18). Strict data security procedures such as encryption and secure API communication are implemented in accordance with industry best practices for health data protection (19) in order to protect sensitive patient information.

### 3.3 Multilingual support and accessibility

Using the Slang package, the system automatically adjusts the language settings to the nation of the clinic identified by the logged-in clinician’s credentials (20). With support for English, Greek, German, Portuguese and Thai, this feature improves accessibility and user convenience by displaying the app in the user’s preferred language.

### 3.4 User interface

**Figure 2:**
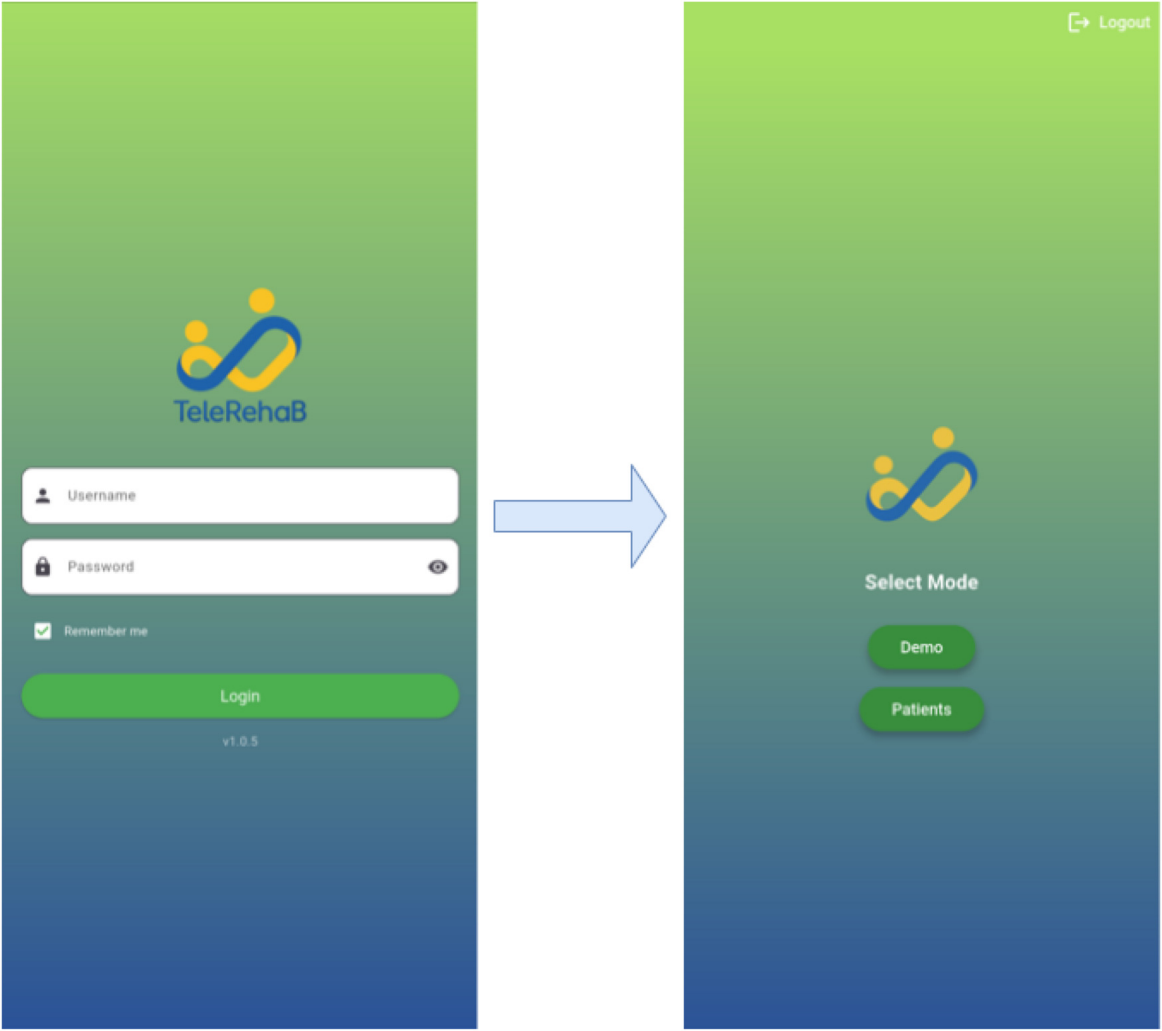
Login interface displaying patient and demo modes.

The app has several screens designed to guide the user through exercises and questionnaires to assess their eHealth literacy. One of the most important screens is the login screen, where clinicians can enter their credentials. Patient mode and demo mode are the two different operating modes that are displayed after logging in, as shown in Figure 2. Each patient in patient mode is linked to a unique patient ID and session, so it is intended for use in a clinical environment (21). On the other hand, in demo mode, no patient-specific credentials are required to test and explore the app. This two-mode system is intended for research or demonstration purposes in addition to practical clinical use. In the Patient Mode, clinicians can see a list of their patients. Each patient can have multiple sessions, with an overview of completed and pending exercises. The interface allows clinicians to select patients and review their progress across different therapy sessions.

### 3.5 Metrics

The selection of metrics to assess digital literacy and motor skills was based on established frameworks and previous research on eHealth literacy (9). Each metric was specifically selected to assess particular aspects of user interaction with the app, ensuring a comprehensive assessment of both digital literacy and practical abilities. The exercises were designed to reflect real-life scenarios that patients might face when using digital health resources, providing relevant insights into their skills. The evaluation intended to provide an expanded overview of the ability to implement eHealth solutions by combining performance-based metrics with self-reported responses.

**Figure 3:**
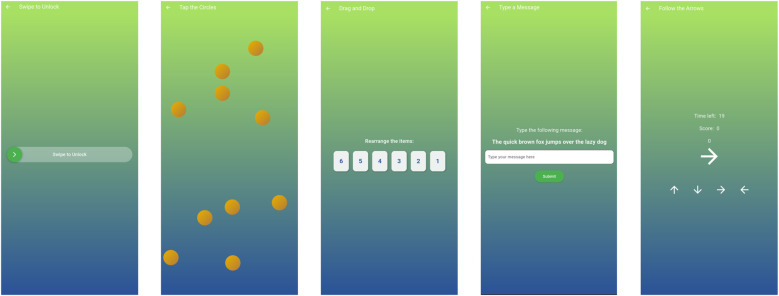
User interface displaying exercises.

Upon selecting a particular patient, the patient is presented with a series of exercises, as displayed in Figure 3 and questionnaires, as displayed in Figure 5. The metrics of both exercises and questionnaires, as being captured by the app, are shown in Table 1.

**Table 1:** Metrics captured by the App.

Exercise/quiz/questionnaire	Metrics	Description
Swipe to unlock	Time to unlock	Time taken for the user to swipe and unlock the screen
Tap the circles	Taps completed, missed taps	Number of circles successfully tapped and missed
Drag and drop	Time to complete	Time taken for the user to correctly rearrange items
Type a message	Time to complete, typing mistakes, time until first backspace, backspace count	Time taken to type the message, number of errors, time until first correction, total corrections made
Follow the arrows	Score, total arrows shown	User’s score and total number of arrows displayed
Badges quiz	Correct responses, response time	Number of correct identifications of badge status and time taken per question
Sessions quiz	Correct responses, response time	Accuracy in interpreting session progress and time taken per calculation
Steps quiz	Correct responses, response time	Accuracy in reading step counter displays and time taken per question
eHEALS questionnaire	Responses	Likert-scale responses assessing eHealth literacy
STAM questionnaire	Responses	Likert-scale responses evaluating attitude towards technology
MDPQ questionnaire	Responses	Responses evaluating mobile device proficiency

The performance-based exercises are designed to assess different aspects of digital literacy and motor skills (22). The “Swipe to Unlock” exercise measures the user’s ability to interact with touchscreen surfaces and records the time it takes to perform the action. The “Tap the Circles” exercise assesses fine motor skills and reaction time, recording both successful and failed touches. The “Drag and Drop” exercise assesses the user’s ability to manipulate objects onthe screen and measures the time it takes to arrange the objects correctly. The “Type a Message” exercise assesses typing skill by measuring time to completion, typing errors, time to first backspace and total number of backspaces. Finally, the “Follow the Arrows” exercise tests the user’s ability to follow the on-screen instructions and records the score and the total number of arrows displayed.

**Figure 4:**
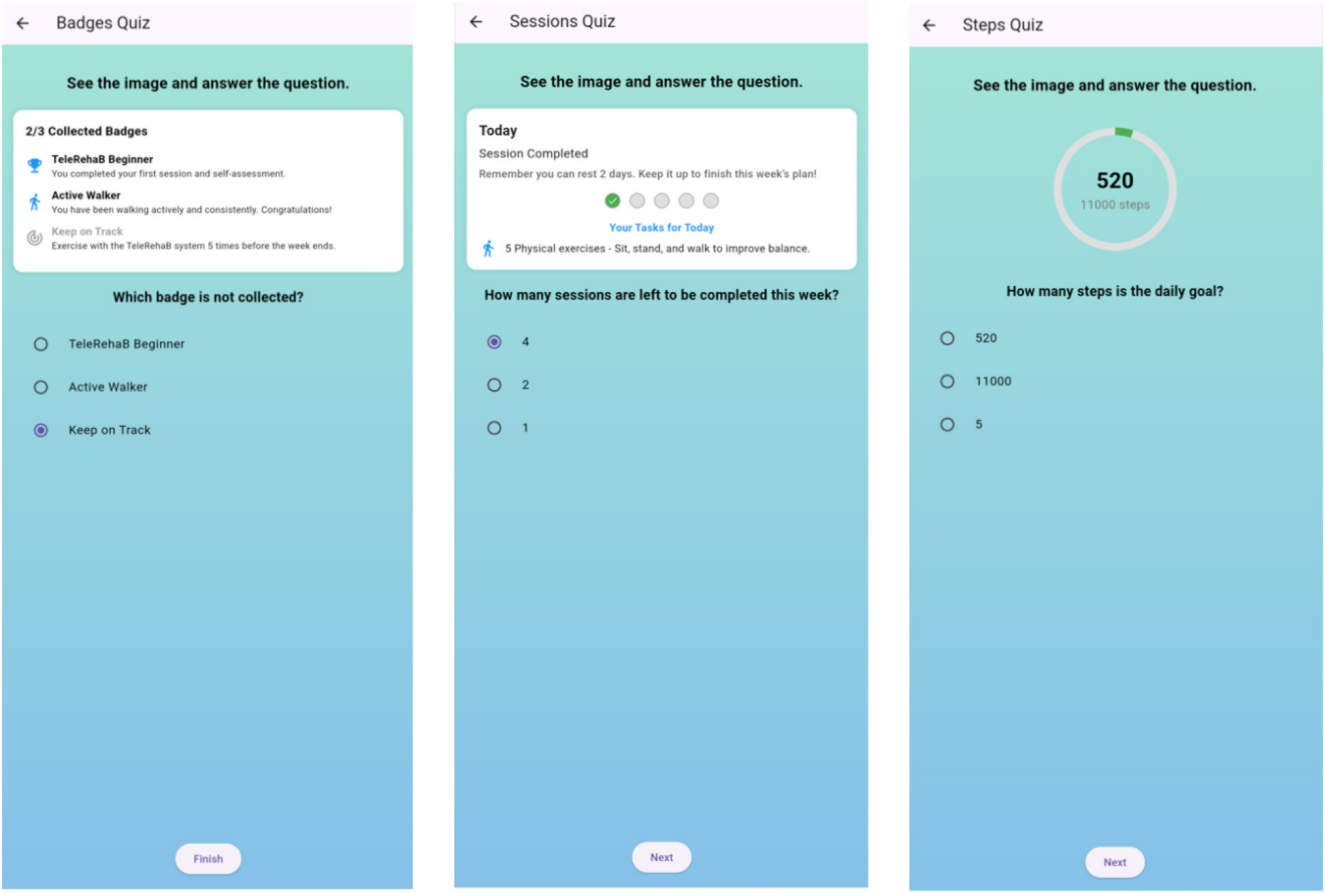
User interface displaying Gamification quizzes: **(a)** badges quiz, **(b)** sessions quiz, **(c)** steps quiz.

In addition to the performance-based exercises, three gamification-focused quizzes were integrated to enhance user engagement and evaluate comprehension of digital health concepts. The “Badges Quiz” evaluates users’ understanding of achievement in systems commonly employed in digital health platforms, by presenting scenarios where users must distinguish between collected and uncollected badges (23). The “Sessions Quiz” assesses the user’s ability to interpret session-based information and track progress over time. It requires users to calculate the remaining sessions based on visual progress indicators (24). The “Steps Quiz” measures the user’s understanding of activity tracking interfaces and tests their ability to read and interpret pedometer displays and daily goals (25). These gamification-based assessments, shown in Figure 4, provide an interactive approach to evaluating users’ ability to navigate common elements found in contemporary digital health applications.

**Figure 5:**
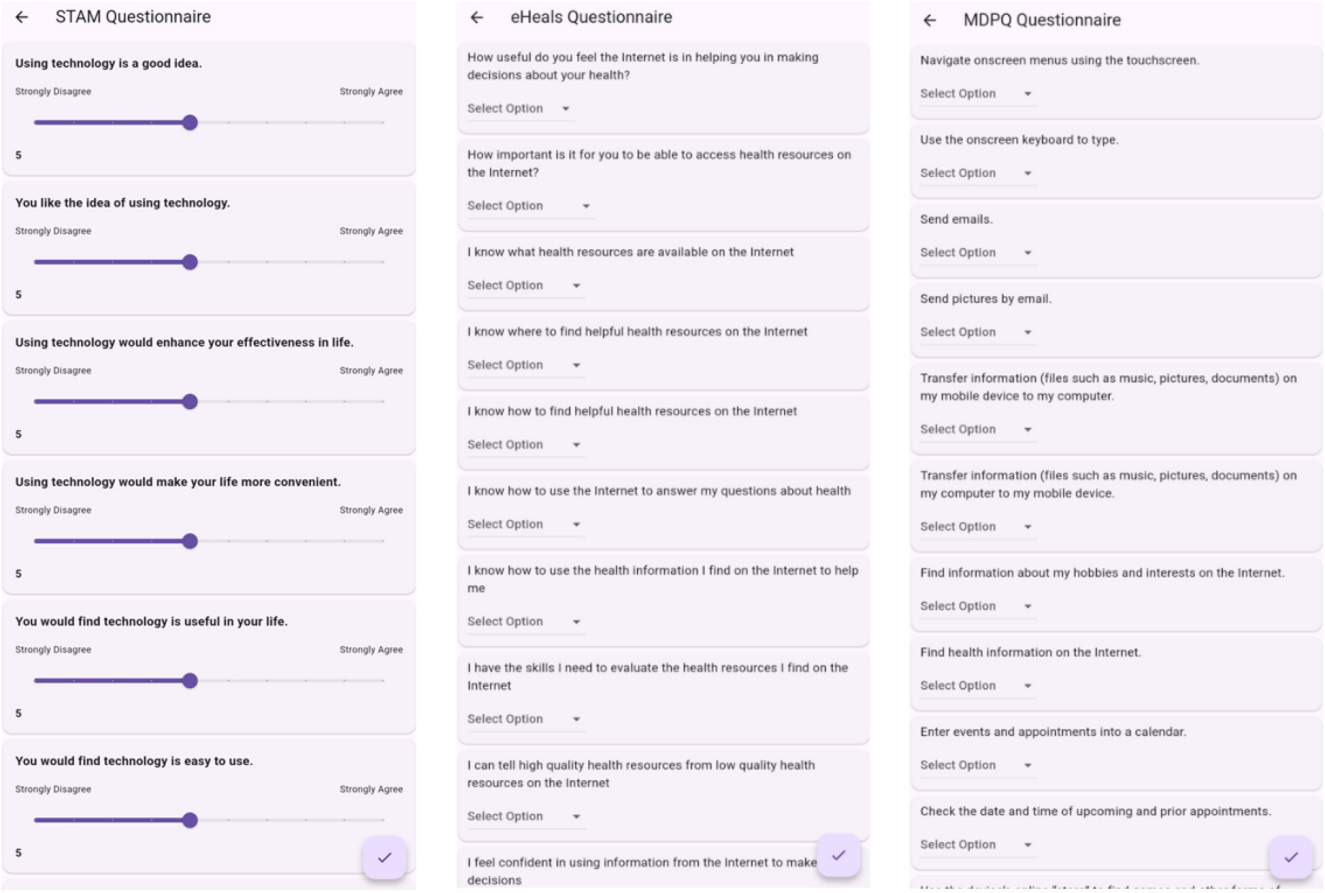
User interface displaying questionnaires.

The integration of questionnaires such as the eHealth Literacy Scale (eHEALS), the Senior Technology Acceptance Model (STAM), and the Mobile Device Proficiency Questionnaire (MDPQ) into digital health literacy tools is critical for enhancing their efficacy and user engagement. These questionnaires, shown in Figure 5, serve a dual purpose: they not only facilitate the customization of digital health resources by evaluating users’ internet utilization patterns, technology acceptance levels, and ability in mobile device operations, but they also contribute to the overall improvement of health literacy. By systematically identifying the unique challenges faced by elders in the adoption of digital technologies, these questionnaires can uncover specific needs and barriers to effective engagement.

The eHEALS questionnaire (26) begins with an introductory question asking users if they use the Internet to find health-related resources. Based on the answer to this question, the app decides whether to proceed with the full eHEALS assessment. This adaptive approach ensures that the assessment is relevant to each user’s digital habits.

The STAM (Senior Technology Acceptance Model) questionnaire (27) was developed to assess older adults’ acceptance of and comfort with using digital health technologies. It assesses the key factors that influence technology acceptance, including perceived usefulness, ease of use and social influence. The questionnaire contains a series of questions on a Likert scale (from 1 to 10) that allow users to indicate their level of agreement or disagreement with statements about their experiences and attitudes towards technology. This assessment is specifically designed for older adults and focuses on variables such as age-related factors and self-efficacy in using digital tools for health-related purposes.

The Mobile Device Proficiency Questionnaire (MDPQ) (28) was integrated to obtain a comprehensive assessment of the user’s ability to perform various tasks on mobile devices. This questionnaire covers a range of activities, from basic operations such as adjusting screen brightness to more complex tasks such as managing app installations and using cloud storage services.

Before each questionnaire is started, the patient is shown an instructions’ screen (28). This screen is designed to provide clear and concise information about the purpose of the questionnaire and instructions on how to complete the questionnaire. For example, before the eHealth Literacy Scale (eHEALS), the introductory screen explains the importance of assessing the user’s confidence in accessing and understanding online health information. Similarly, before the Senior Technology Acceptance Model (STAM) and the Mobile Device Proficiency Questionnaire (MDPQ), the user gets informed of the specific objectives of the questionnaires, that might involve evaluating the benefits of using mobile devices or measuring the usability of the equipment (29).

### 3.6 Sessions’ functionality

Throughout the course of treatment, clinicians can track progress and changes in eHealth literacy according to the app’s support during numerous sessions (30, 31). A new session begins or an incomplete one is carried over, each time a patient finishes a series of exercises and questionnaires. This feature acknowledges potential weariness or time limits of older individuals using digital tools by allowing assessments to be resumed after interruption.

When an internet connection is available, the Hive-stored session data is synchronized with the MariaDB database on the server. Healthcare professionals always have access to the most recent data regarding the status of their patients due to this synchronization (32). The methodology by which the sessions are organized also makes it easier to create progress reports, which helps doctors evaluate patient outcomes as they progress.

On the other hand, while an application’s Demo mode is intended for one-time use or demonstration, it does not make use of the session process. The app’s adaptability in meeting clinical and instructional demands is highlighted by the difference in session management between the demo and patient modes.

### 3.7 Deployment and platform availability

The eHealth Literacy App is distributed to clinical sites through controlled installation packages for both Android and iOS devices. This deployment strategy ensures that only authorized clinical sites receive the application and enables consistent version management across all installations. The backend system is hosted centrally at the Biomedical Engineering Laboratory, NTUA, Athens, as part of the overall TeleRehaB DSS project infrastructure. Authentication is managed through clinician credentials, so that only authorized healthcare professionals within the TeleRehaB project can access the system. This centralized model simplifies maintenance while maintaining security protocols for handling sensitive patient data.

## 4 Results

The eHealth Literacy Application was made available to the organization’s internal users in the first test phase. This group included employees with different functions and technical knowledge. The pre-pilot phase was used to evaluate the usability and overall user experience of the app before the pilot phase for patients initialized. The software was used by internal users who answered the questionnaires, completed the exercises, and performed the gamification quizzes. This diverse group provided comprehensive usability by providing insightful feedback on the app’s performance in terms of various technical backgrounds, interface design and navigation.

### 4.1 Usability and functionality testing

Usability of the eHealth Literacy App was assessed using the System Usability Scale (SUS) (33), a widely recognized tool that provides a reliable measure of user satisfaction and ease of use. After interacting with the app, participants completed the SUS (34), which consists of ten items that evaluate various aspects of usability. This approach allows for quantitative analysis of user feedback, offering insights into the app’s overall effectiveness and areas for improvement (35). The SUS scores were subsequently analyzed to identify strengths and weaknesses in the user interface and overall experience.

The diverse group of internal users could be valuable sources of perspective regarding usability, interface design, and general user experience, even if they are not the targeted audience. It was expected that their comments would indicate weaknesses related to navigation, engagement, or the flow of interaction processes in the app. The focus of this study was to assess how well the participants would be able to perform the set exercises, complete the posed questions, respond to the gamification quizzes, as well as to assess the connectivity between the local database (Hive) and the backend APIs for session data storage and synchronization.

The data gathered through this testing phase were mostly aimed at the time taken to perform each exercise, the number of completed exercises and opinions regarding the app’s features. In addition, the participants were invited to submit open-ended comments on the transparency of the instructions, the app’s navigation and interactivity during live performance (36). Furthermore, the data connected with how well the multilingual feature (using Slang) operated was also noted.

Insights gained from this phase enabled improvement of particular features of the app. In particular, the content of the introductory screens that accompanied each questionnaire was modified, as well as translation of some terminologies, based on user comments. Such observations will constitute the foundation for the full pilot study where the application will be used with real patients in a clinical setting.

This phase established that the application is technically sound, as data was managed seamlessly between the local device and Hive storage and the backend server. The testing subjects involved lost scenarios when the internet was available for the first portion of the session but ceased for the latter section of it. This was done in three variations; whenever the device was disconnected from the network, the data stored locally on it was always synchronized with the server once the connection was restored. Such validation steps allow for a usage scenario where an application will run in environments where internet connection is poor and data loss would otherwise occur.

### 4.2 System stability

The REST APIs created in C# language for communication between the application and the backend server were successfully completed within an average data exchange time of less than 1 s under stable internet connections. With the help of these APIs, patient records can be updated immediately, allowing clinicians to keep track of patients’ needs and modify the treatment if necessary (37).

### 4.3 Assesment of risk of bias

Potential sources of bias in the evaluation of the eHealth Literacy App were thoroughly addressed during the internal testing phase. We considered selection bias in participant recruitment, potential inconsistencies in exercise administration, and limitations of self-reported data. To mitigate these concerns, we included internal users with varying technical backgrounds, standardized testing protocols, and compared self-reported assessments with observed performance. This internal testing was primarily designed to validate technical functionality rather than comprehensively evaluate eHealth literacy outcomes. These preliminary findings have strengthened the app’s design, preparing it for the upcoming multicentric clinical study where comprehensive quantitative data will be collected from 230 patients comprising the intervention arm of the study.

## 5 Discussion

Within the TeleRehaB DSS project, the development and pilot study of the eHealth Literacy App demonstrates its relevance in overcoming the existing digital and eHealth literacy challenges of patients receiving balance physiotherapy. These issues have been resolved through eHealth literacy, which has been shown to enable patients to participate appropriately in health interventions delivered via the internet, directly impacting adherence to a prescribed treatment regime. The use of an app that assesses the level of digital and eHealth literacy of patients helps healthcare providers to develop appropriate management strategies to avoid patients having difficulties in using the digital platforms that are helpful to their rehabilitation.

The implementation of all of the above, including the app’s exercises, self-reported questionnaires, and gamification quizzes, illuminates the readiness of technologically literate patients to use technology for their health. This combination of self-reported ease, practice-based competence, and interactive assessments explores a key limitation of existing digital health solutions where patients self-assess ’comfort’ with technology without clearly outlining basic skills.

The inclusion of gamification-oriented quizzes (Badges Quiz, Sessions Quiz, and Steps Quiz) fills a critical gap in existing assessments of eHealth literacy by assessing users’ understanding of the interaction mechanisms common in modern digital health platforms. These elements are becoming increasingly important as health apps employ game-like features to improve user motivation and adherence to treatment protocols. The quizzes showed that participants could effectively interpret visual progress indicators and success systems. This suggests that gamification elements, when properly designed, do not present additional barriers to target audience engagement with digital health applications. This finding is particularly relevant for the TeleRehaB DSS project, as the integration of motivational elements through gamification can potentially improve patients’ long-term engagement in rehabilitation protocols.

During the testing phase involving internal users, it was noted that session data was not lost in even the hardest of conditions thanks to Hive which maintained local data storage even in the absence of a stable internet connection. This is an important factor in remote or low-resource settings where internet connection is very unreliable and data integrity remains important. The use of Slang for bringing automatic language adaptation through the region of the doctor’s clinic also worked well as it made the application more usable in different languages and cultures. This functionality compliments the aim of increasing engagement and participation especially of otherwise underserved patients in such digital health interventions.

The application was built with dual-mode functionality which is one of the most useful features of the application as it allows for use by self-identified patients in specific sessions or in anonymous demo. This offered convenience to clinicians, as the app can be used in different patient rehabilitation scenarios or purely educational or training aspects. Further, the ability to perform multiple sessions per patient allows the clinicians to have more information concerning the patients over time and make better decisions regarding their further treatment and the interventions that need to be made.

When comparing our approach to existing work in eHealth literacy assessment, our study offers several novel contributions to the field. Unlike tools such as eHEALS (2) that primarily rely on self-reported capabilities, our eHealth Literacy App integrates performance-based exercises, gamification quizzes, and standardized questionnaires, addressing the need for real-world performance assessment. Pavan et al. (12) investigated robotic and non-robotic tele-rehabilitation models and demonstrated significant recovery of cognitive and motor function in stroke patients. Bok et al. (13) focused on home-based high-tech rehabilitation programs and highlighted the effectiveness of virtual reality in improving upper limb recovery. Jaramillo-Isaza et al. (14) investigated the use of wearable sensors and emphasized their role in providing real-time feedback to improve patient adherence to therapy. In contrast to these studies, our research introduces the eHealth Literacy App, which not only incorporates elements of these innovative technologies, but also prioritizes eHealth literacy and enables patients to effectively engage with digital health interventions. Furthermore, while (15) identified the importance of performance-based assessments, our work extends this finding by creating a comprehensive framework that translates assessment results directly into personalized treatment recommendations—a capability not present in existing eHealth literacy tools. Additionally, the incorporation of automated multilingual support distinguishes our approach from existing solutions, directly addressing the cultural and linguistic barriers highlighted by (9) as determinants of health disparities. Finally, through dual-mode functionality and the integration of self-assessments, our study presents a comprehensive solution that has the potential to improve both patient engagement and treatment adherence in rehabilitation settings.

### 5.1 Limitations and future perspectives

The application had practical exercise-based assessments that measured motor and cognitive skills relevant to the proficiency of digital literacy. However, some changes would enhance the interface, primarily the start up screens of the questionnaires. Additionally, the current implementation assumes relatively stable network connectivity for optimal data synchronization, which may present challenges in some clinical environments. These limitations emphasize the importance of progressive enhancement of the app to ensure it achieves its objectives whilst being simple and easy to use.

Moreover, while the initial results are promising, there are limitations to the current findings as the tests were conducted in a controlled organizational setting and not with the patient population intended for the app. As the pilot study progresses and the app is used in a clinical setting, further evaluation will be required to assess the impact of the app on patient adherence, engagement and health outcomes in practice. The upcoming clinical validation will provide more comprehensive insights into the effectiveness and scalability of the app.

### 5.2 Conclusions

In conclusion, the eHealth Literacy App developed as part of the TeleRehaB DSS project, represents a significant advancement in addressing the digital and eHealth literacy of patients undergoing balance physiotherapy. By incorporating self-assessments, performance-based exercises, and interactive gamification quizzes, the app provides a comprehensive assessment of a patient’s digital skills and enables healthcare providers to develop tailored management strategies. The results of the initial testing phase show that the app is technically robust, well received by users and adaptable to different cultural contexts. In addition, the dual-mode functionality improves accessibility, allowing both self-identified patients and anonymous users to benefit from the app’s features. Ultimately, this innovative approach is expected to enhance patient adherence to rehabilitation protocols and could contribute to better health outcomes, highlighting the importance of integrating technology into healthcare. Future studies will further expand on these findings by evaluating the app’s effectiveness in real-world clinical settings, which may pave the way for improved patient care in an increasingly digital healthcare landscape.

## Data Availability

The datasets presented in this article are not readily available because still on process/under project progress. Requests to access the datasets should be directed to kostasgeo@biomed.ntua.gr.

## References

[1] Nutbeam D. The evolving concept of health literacy. Social Sci Med. (2008) 67(12):2072–8. 10.1016/j.socscimed.2008.09.050 18952344

[2] Norman CD Skinner HA. eHealth literacy: essential skills for consumer health in a networked world. J Med Internet Res. (2006) 8(2):e9. 10.2196/jmir.8.2.e9 16867972 PMC1550701

[3] El Benny M Kabakian-Khasholian T El-Jardali F Bardus M. Application of the ehealth literacy model in digital health interventions: scoping review. J Med Internet Res. (2021) 23(6):e23473. 10.2196/23473 34081023 PMC8212628

[4] Mulukuntla S. Digital health literacy: empowering patients in the era of electronic medical records. EPH Int J Med Health Sci. (2020) 6(4):23–30. 10.53555/eijmhs.v6i4.210

[5] Landry KE. Using eHealth to improve health literacy among the patient population. Creat Nurs. (2015) 21(1):53–7. 10.1891/1078-4535.21.1.53 25842526

[6] Lequerica AH Donnell CS Tate DG. Patient engagement in rehabilitation therapy: physical and occupational therapist impressions. Disabil Rehabil. (2009) 31(9):753–60. 10.1080/09638280802309095 19034722

[7] Dijkman EM Ter Brake WW Drossaert CH Doggen CJ. Assessment tools for measuring health literacy and digital health literacy in a hospital setting: a scoping review. Healthcare. (2024) 12:11. 10.3390/healthcare12010011 PMC10778720 38200917

[8] Lee J Tak SH. Factors associated with eHealth literacy focusing on digital literacy components: a cross-sectional study of middle-aged adults in South Korea. Digit Health. (2022) 8:20552076221102765. 10.1177/20552076221102765 35615270 PMC9125061

[9] Arias López MDP Ong BA Borrat Frigola X Fernández AL Hicklent RS Obeles AJT, et al. Digital literacy as a new determinant of health: a scoping review. PLOS Digit Health. (2023) 2(10):e0000279. 10.1371/journal.pdig.0000279 37824584 PMC10569540

[10] Lee J Lee E-H Chae D. eHealth literacy instruments: systematic review of measurement properties. J Med Internet Res. (2021) 23(11):e30644. 10.2196/30644 34779781 PMC8663713

[11] Van Der Vaart R Drossaert C. Development of the digital health literacy instrument: measuring a broad spectrum of health 1.0 and health 2.0 skills. J Med Internet Res. (2017) 19(1):e27. 10.2196/jmir.6709 28119275 PMC5358017

[12] Pavan A Fasano A Lattanzi S Cortellini L Cipollini V Insalaco S, et al. Effectiveness of two models of telerehabilitation in improving recovery from subacute upper limb disability after stroke: robotic vs. non-robotic. Brain Sci. (2024) 14(9):941. 10.3390/brainsci14090941 39335435 PMC11430637

[13] Bok S-K Song Y Lim A Jin S Kim N Ko G. High-tech home-based rehabilitation after stroke: a systematic review and meta-analysis. J Clin Med. (2023) 12(7):2668. 10.3390/jcm12072668 37048751 PMC10095213

[14] Jaramillo-Isaza S Delis AL Herrera EP Ruiz-Olaya AF. Enhancing telerehabilitation using wearable sensors and AI-based machine learning methods. In: *Computational Approaches in Biomaterials and Biomedical Engineering Applications*. CRC Press (2024). p. 266–98.

[15] Xie L Mo PK. Comparison of eHealth literacy scale (eHEALS) and digital health literacy instrument (DHLI) in assessing electronic health literacy in Chinese older adults: a mixed-methods approach. Int J Environ Res Public Health. (2023) 20(4):3293. 10.3390/ijerph20043293 36833987 PMC9967021

[16] Windmill E. Flutter in Action. Shelter Island, NY: Manning Publications (2019).

[17] Richter J. Applied Microsoft. NET Framework Programming. Redmond: Microsoft Press (2002). Vol. 1.

[18] Capriolo E Wampler D Rutherglen J. Programming Hive. Sebastopol, CA: O’Reilly Media, Inc. (2012).

[19] Délétroz C Allen MC Sasseville M Rouquette A Bodenmann P Gagnon M-P. eHealth literacy measurement tools: a systematic review protocol. Syst Rev. (2022) 11(1):205. 10.1186/s13643-022-02076-2 36151577 PMC9508732

[20] Sun Z Zemel R Xu Y. A computational framework for slang generation. Trans Assoc Comput Linguist. (2021) 9:462–78. 10.1162/tacl/a/00378

[21] Brørs G Larsen MH Hølvold LB Wahl AK. eHealth literacy among hospital health care providers: a systematic review. BMC Health Serv Res. (2023) 23(1):1144. 10.1186/s12913-023-10103-8 37875882 PMC10599073

[22] Lê M Quémart P Potocki A Gimenes M Chesnet D Lambert E. Modeling the influence of motor skills on literacy in third grade: contributions of executive functions and handwriting. PLoS One. (2021) 16(11):e0259016. 10.1371/journal.pone.0259016 34843490 PMC8629244

[23] Floryan M Chow PI Schueller SM Ritterband LM. The model of gamification principles for digital health interventions: evaluation of validity and potential utility. J Med Internet Res. (2020) 22(6):e16506. 10.2196/16506 32519965 PMC7315368

[24] Lin Z Althoff T Leskovec J. I'll be back: on the multiple lives of users of a mobile activity tracking application. In: International World Wide Web Conferences Steering Committee, editor. Proceedings of the 2018 World Wide Web Conference (WWW '18), April 23–27, 2018, Lyon, France. New York, NY: ACM (2018). p. 1501–11. 10.1145/3178876.3186062 PMC5959281 29780978

[25] Rye Hanton C Kwon Y-J Aung T Whittington J High RR Goulding EH Mobile phone-based measures of activity, step count, and gait speed: results from a study of older ambulatory adults in a naturalistic setting. JMIR Mhealth Uhealth. (2017) 5(10):e104. 10.2196/mhealth.5090 28974482 PMC5645644

[26] Gazibara T Cakic J Cakic M Pekmezovic T Grgurevic A. eHealth and adolescents in serbia: psychometric properties of eheals questionnaire and contributing factors to better online health literacy. Health Promot Int. (2019) 34(4):770–8. 10.1093/heapro/day028 29800141

[27] Chen K Chan AHS. Gerontechnology acceptance by elderly Hong Kong Chinese: a senior technology acceptance model (STAM). Ergonomics. (2014) 57(5):635–52. 10.1080/00140139.2014.895855 24655221

[28] Roque NA Boot WR. A new tool for assessing mobile device proficiency in older adults: the mobile device proficiency questionnaire. J Appl Gerontol. (2018) 37(2):131–56. 10.1177/0733464816642582 27255686 PMC9394541

[29] Shaw Jr G Castro BA Gunn LH Norris K Thorpe Jr RJ. The association of eHealth literacy skills and mHealth application use among us adults with obesity: analysis of health information national trends survey data. JMIR mHealth uHealth. (2024) 12:e46656. 10.2196/46656 38198196 PMC10809169

[30] Bevilacqua R Strano S Di Rosa M Giammarchi C Cerna KK Mueller C, et al. eHealth literacy: from theory to clinical application for digital health improvement. Results from the access training experience. Int J Environ Res Public Health. (2021) 18(22):11800. 10.3390/ijerph182211800 34831555 PMC8618977

[31] Kocher A Simon M Dwyer AA Blatter C Bogdanovic J Künzler-Heule P, et al. Patient and healthcare professional eHealth literacy and needs for systemic sclerosis support: a mixed methods study. RMD Open. (2021) 7(3):e001783. 10.1136/rmdopen-2021-001783 34475248 PMC8413951

[32] Cramer A Keinki C Saur F Walter S Hübner J. eHealth literacy, internet and eHealth service usage: a survey among a German municipality. J Public Health. (2023) 33(2):237–48. 10.1007/s10389-023-01997-z

[33] Martins AI Rosa AF Queirós A Silva A Rocha NP. European portuguese validation of the system usability scale (SUS). Procedia Comput Sci. (2015) 67:293–300. 10.1016/j.procs.2015.09.273

[34] Lewis JR. The system usability scale: past, present, and future. Int J Hum Comput Interact. (2018) 34(7):577–90. 10.1080/10447318.2018.1455307

[35] Cheah W-H Jusoh NM Aung MMT Ab Ghani A Rebuan HMA. Mobile technology in medicine: development and validation of an adapted system usability scale (SUS) questionnaire and modified technology acceptance model (TAM) to evaluate user experience and acceptability of a mobile application in MRI safety screening. Indian J Radiol Imaging. (2023) 33(1):036–45. 10.1055/s-0042-1758198 PMC9968523 36855734

[36] Fu B Lin J Li L Faloutsos C Hong J Sadeh N. Why people hate your app: making sense of user feedback in a mobile app store. In: *Proceedings of the 19th ACM SIGKDD International Conference on Knowledge Discovery and Data Mining* (2013). p. 1276–84.

[37] Hamad H Saad M Abed R. Performance evaluation of restful web services for mobile devices. Int Arab J Inf Technol. (2010) 1(3):72–8.

